# Implementation of a medicine management plan (MMP) to reduce medication-related harm (MRH) in older people post-hospital discharge: a randomised controlled trial

**DOI:** 10.1186/s12877-022-03555-w

**Published:** 2022-11-11

**Authors:** Khalid Ali, Ekow A. Mensah, Eugene Ace McDermott, Frances A. Kirkham, Jennifer Stevenson, Victoria Hamer, Nikesh Parekh, Rebekah Schiff, Tischa Van Der Cammen, Stephen Nyangoma, Sally Fowler-Davis, Graham Davies, Heather Gage, Chakravarthi Rajkumar

**Affiliations:** 1 University Hospitals-Sussex NHS Trust, Brighton, UK; 2 Brighton and Sussex Medical School, University of Sussex, Brighton, UK; 3 Institute of Pharmaceutical Science, King’s College London, London, UK; 4 Honorary Patient and Public Involvement (PPI) Fellow, Centre for Applied Health and Social Care Research, Kingston University, Kingston, UK; 5 St Thomas’ Hospital, London, UK; 6 Department of Human-Centered Design, Faculty of Industrial Design Engineering, Delft University of Technology, Delft, The Netherlands; 7 Department of Internal Medicine, Section of Geriatric Medicine, Erasmus University Medical Center, Rotterdam, The Netherlands; 8 Imperial College-London, London, UK; 9 Advanced Wellbeing Research Centre- Sheffield Hallam University, Sheffield, UK; 10 Department of Health Economics, University of Surrey, Guildford, UK

**Keywords:** Medication, Harm, Older people, Risk, Prediction, Model, Hospital, Discharge, Cost

## Abstract

**Background:**

Medication-related harm (MRH) is an escalating global challenge especially among older adults. The period following hospital discharge carries high-risk for MRH due to medication discrepancies, limited patient/carer education and support, and poor communication between hospital and community professionals. Discharge Medical Service (DMS), a newly introduced NHS scheme, aims to reduce post-discharge MRH through an electronic communication between hospital and community pharmacists. Our study team has previously developed a risk-prediction tool (RPT) for MRH in the 8-weeks period post discharge from a UK hospital cohort of 1280 patients. In this study, we aim to find out if a Medicines Management Plan (MMP) linked to the DMS is more effective than the DMS alone in reducing rates of MRH.

**Method:**

Using a randomized control trial design, 682 older adults ≥ 65 years due to be discharged from hospital will be recruited from 4 sites. Participants will be randomized to an intervention arm (individualised medicine management plan (MMP) plus DMS) or a control arm (DMS only) using a 1:1 ratio stratification. Baseline data will include patients’ clinical and social demographics, and admission and discharge medications. At 8-weeks post-discharge, a telephone interview and review of GP records by the study pharmacist will verify MRH in both arms. An economic and process evaluation will assess the cost and acceptability of the study methods.

**Data analysis:**

Univariate analysis will be done for baseline variables comparing the intervention and control arms. A multivariate logistic regression will be done incorporating these variables. Economic evaluation will compare the cost-of-service use among the study arms and modelled to provide national estimates. Qualitative data from focus-group interviews will explore practitioners’ understanding, and acceptance of the MMP, DMS and the RPT.

**Conclusion:**

This study will inform the use of an objective, validated RPT for MRH among older adults after hospital discharge, and provide a clinical, economic, and service evaluation of a specific medicines management plan alongside the DMS in the National Health Service (UK).

**Supplementary Information:**

The online version contains supplementary material available at 10.1186/s12877-022-03555-w.

## Background

Medication Without Harm is the World Health Organisation’s (WHO) Third Global Patient Safety Challenge. It aims to “reduce the level of severe, avoidable harm related to medication by 50% over 5 years, globally” [1]. Medication-related harm (MRH) includes harm from adverse drug reactions (ADR), non-adherence and medication errors [2].

In a large, prospective UK study of 18,820 patients, the prevalence of an ADR-related admission was 6.5% with the average age of 76 years old and an estimated cost of £466 million annually [3]. In another observational study in Rotterdam, 24% of admissions in over 70 year olds were due to ADRs [4]. A large, retrospective study of adverse events (AE) across 58 US hospitals identified 5077 cases of inpatient admissions in the over 65 s from 99,628 emergency department visits with half occurring in those older than 80 [5]. A meta-analysis shows elderly patients are four times more likely to be admitted with an ADR compared to younger patients [6]. In a systematic review of MRH in older adults, between 17–51% patients experience MRH within 30 days post-discharge [2, 7]. Our study team have shown that in the 8 weeks following hospital discharge, 37% of over 65 s experienced MRH with an estimated cost of £400million annually [8].

Factors contributing to MRH in older people are multiple including multimorbidity and polypharmacy [9], age-related changes in pharmacokinetics and pharmacodynamics [10] and non-adherence to medications [11]. Transition of care at hospital discharge is high-risk period for the occurrence of MRH with multiple contributory factors: the impact of acute illness, an inpatient stay and patient deconditioning [12], medication discrepancies at admission or discharge [13], patient/carer education on discharge medication regimens, the use of new medications [14], and poor communication between secondary and primary care [15, 16].

The WHO identifies three key target areas to protect patients from MRH: high-risk situations, polypharmacy, and transitions of care [1]. The WHO defines high-risk situations as certain clinical circumstances in which the impact of MRH may be greater; this includes older individuals and those with hepatic or renal impairment [17]. Discharge of an older person is an especially high-risk situation encompassing many factors that might lead to MRH.

The NHS Discharge Medicines Service (DMS) is a newly commissioned community pharmacy service aiming to reduce avoidable post-discharge MRH based on an initiative led and delivered by the Academic Health Science Networks (AHSNs). The DMS is a system of electronic communication allowing hospital pharmacists to refer patients to community pharmacists to ensure that patients receive adequate support post-discharge. The evidence for the DMS is informed by several studies showing reduced hospital readmission rates and shorter hospital stay [18–21].

Although effective, patient selection for the NHS DMS is based on hospital pharmacists’ judgement and not on any evidence-based risk stratification data [22]. NHS England supports the adoption of risk stratification using risk prediction models (RPMs) to identify individuals who will derive the most benefit from target interventions [23]. Risk stratification is increasingly important in a healthcare system challenged by an ageing population [24], and an increasing prevalence of healthcare use due to MRH [25].

Six RPMs exist: McElnay [26], the BADRI Model [27], the GerontoNet ADR risk score [28], Trivalle [29], the PADR-EC score [30], and the ADRROP prediction scale [31]. These RPMs do not predict the risk of MRH occurring in the post-hospital discharge period. No impact studies of these tools have been published. They predict the risk of inpatient ADR/ADE [27, 29, 31, 32] or risk of an unplanned admission being due to ADR/ADE [30, 33].

Our study team has conducted the PRIME (Prospective study to develop a model to stratify the RIsk of Medication-related harm in hospitalised Elderly patients) study and developed and internally validated the first RPT to predict the absolute risk of an older person experiencing MRH in the 8-week post-discharge period [34]. The RPT was developed though a large multicentre, prospective observational cohort study. The PRIME RPT consists of eight variables routinely collected in hospital (age, gender, antiplatelet drug, sodium level, antidiabetic drug, past ADR history, number of medicines, and living alone). This tool considers demographic, medical and social factors in predicting the absolute risk of MRH. The PRIME tool calculates risk of a definite MRH; MRH that was classified as’probable’ or’possible’ was excluded.

Currently, there are no tools in clinical practice to target interventions to high-risk patients in the community following hospital discharge. The PRIME tool is the first RPT able to predict risk of MRH in older adults in the community in the 8 weeks post-discharge.

## Rationale

Medication-related harm (MRH) for this study will include adverse drug reactions, medication errors, and a failure to take/receive medication, either following non-adherence or a failure in the supply chain.

The WHO identifies transition of care as a key area when it is necessary to protect patients from harm [1]. Previous interventions in the post-discharge period have been ineffective [35–38]. Published evidence for the NHS DMS has shown that the DMS can decrease readmission rate and shorten the length of hospital stay [18–21]. However, patient selection for DMS is based on hospital pharmacists’ judgement and not on any evidence-based risk stratification data [22]. Our study team has shown that clinical judgement alone cannot predict MRH in older patients [39].

NHS England recognises that risk stratification using RPMs can target those at greatest risk and those who are most likely to derive benefit from intervention [23]. The PRIME tool is the first tool to predict absolute risk of MRH in the 8 weeks post-discharge period and is a better predictor than clinical judgement [39]. A medicine management plan has been developed by the research team in consultation with patients, carers, and healthcare professionals.

MRH risk prediction tools are not routinely used in clinical practice, as these tools have not been assessed for impact and implementation [40]. The PRIME tool has been transparently developed and internally validated. To satisfy the next stage of risk-prediction model creation, the impact of the tool will be assessed on a new sample of older individuals. Targeted interventions at high-risk individuals may be a clinically and cost-effective intervention in reducing the rates of MRH in older adults. The PRIME team in collaboration with the AHSN-KSS will implement a risk-stratification approach linked with the NHS DMS. The study will recruit patients aged 65 and older discharged from 4 NHS trusts. The control arm will consist of NHS DMS care only. The intervention arm will consist of NHS DMS in addition to a specific medicines management plan (MMP) developed by the study team in consultation with patients and carers. The MMP will consist of:

1. A copy of the hospital discharge summary
2. Specific education about the benefits of their medications and possible medication-related harm from discharge medications. Education will be delivered by the ward pharmacist and / or the ward doctor to the intervention participants (and family member/carers/friend if available) at the point of discharge.
3. Clear guidance on who study intervention participants and their carer/family member/friend can contact (the GP or the community pharmacist) if patients experience any MRH.
4. The name and contact of the community pharmacist will be provided by the ward pharmacist to those in the intervention arm/their family member/carer/friend.
5. A copy of the percentage/ probability of harm from discharge medications calculated using the PRIME study RPT. The risk score will be presented in a visual analogue scale and will be offered to intervention patients and (if available their family member/carer/friend). *See* Appendix *fo*r *documents.*

## Research question/aim

### Research question

Will a Medicines Management Plan linked to the NHS DMS be more effective than the NHS DMS alone in reducing rates of MRH?

### Objectives

I. To measure and compare the rates of MRH in the two groups.
II. To measure the costs of delivering the intervention and any associated MRH-related service use in the two groups in the 8-week study period, and to perform modelling to provide national cost estimates.
III. To undertake a process evaluation.

### Outcome

A clinical, economic, and service evaluation of NHS DMS alone compared to a MMP linked with the NHS DMS in reducing MRH.

## Study methods and design

Study participants will be recruited from 4 hospital sites: Royal Sussex County Hospital in Brighton, Ashford and St Peter’s Hospital in Ashford, Medway Hospital in Kent, and Royal Devon Hospital in Exeter. The proposed study is a Randomised Control Trial (RCT), as patients will be randomised into either the NHS DMS alone (control arm/standard care), or RPT-stratification plus MMP linked to the NHS DMS (intervention arm).

### Sample size determination

In determining the sample size, the proportion of study participants developing MRH when given standard care will be labelled as p_1_, while the proportion of study participants developing MRH in intervention arm will be labelled p_2_. Common measures for comparing whether the two proportions are statistically significantly different are:

1. Difference, $$\delta ={p}_{1}-{p}_{2}$$
2. Odds-ratio, $$\Psi =\frac{{p}_{1}/\left(1-{p}_{1}\right)}{{p}_{2}/\left(1-{p}_{2}\right)}$$

A systematic review of MRH in older adults found that between 17 and 51% of patients’ experience MRH within 30 days of hospital discharge [2]. In the UK, approximately 28% of older adults (≥ 65 years) use health services due to MRH within the 8 weeks following hospital discharge [8]. Therefore, our choice of the prevalence in the sample size calculation is limited to MRH rates between 20—40%. We take the prevalence of MRH among the group under standard treatment to be 35% and an odds-ratio of 1.6 is considered large enough to result in a clinically important difference in MRH rates between arm 1 and arm 2. These choices of *p*_*1*_ and odds-ratio corresponds to δ = 0.098.

To determine the required sample size to estimate a difference δ that is clinically relevant, the method of Fleiss, Tytun, and Ury [41] was used. This method has been implemented in the function “bsamsize” given in R package “Hmisc” [42]. To calculate sample size using this method, one needs to provide the following parameters: the prevalence of MRH among people given standard treatment ($p$_*1*_) and among people given the new care/intervention ($p$ 2), odds-ratio (*Ψ)*, the statistical power we wish to achieve, and the margin of error.

We found that a sample size of *n* = 682 (341 participants on each arm) will be required to detect δ = 0.098 (i.e., odds-ratio = 1.6), with 80% statistical power, and a 5% margin of error, if we assume that the prevalence of MRH among those on standard treatment is 35%.

### Sampling technique

After enrolment, study participants will be randomised to either the intervention arm or the control arm receiving standard treatment: a 1-to-1 ratio stratified by site, with allocations permuted between blocks. The randomisation sequence will be created using the R software package [42]: “randomizeR”, a program that generates randomized equi-probable sequences through a procedure called Permuted Block Randomization (PBR) with block constellation.

#### Recruitment

In each hospital, the study research nurse, liaising with the medical team on the wards, will approach patients aged 65 years and over due to be discharged in the next 48 h. We aim to recruit six patients each week in view of the ease of screening and recruiting study participants. Consequently, we will be able to recruit 682 patients to the study from the 4 sites in approximately 7.5 months.

#### Obtaining consent

Potential study participants will be provided with a participant information sheet and the research nurse will explain the study to them. They will be given the opportunity to ask questions, and the time to consider their participation up until the point of discharge. Informed written consent will be sought.

If a potential participant lacks capacity to consent, a family member/friend/carer will be asked to act as a personal consultee and to support the potential participant taking part in the study. If the potential participant regains capacity prior to discharge, they will be invited to take part in the study. It is important to include those who lack capacity, as we do not want to exclude those who are most likely to experience MRH i.e. those most vulnerable due to frailty and/or cognitive limitation. If a potential participant lacks capacity and a suitable personal consultee is not available, they will not be included in the study. Continued consent will be assumed throughout the 8-week study period. Participants with capacity will be asked to provide a family member//friend/carer to contact, in case that they lose their capacity if they are re-admitted or at the 8-week phone call. This approach will be needed in case that the family member//friend/carer needs to be contacted, to act as a consultee to consent for the patient who has lost capacity for continued participation. The patient will be withdrawn if there is no consultee, if the consultee decides to withdraw the individual from the study or if the participant themselves wishes to be withdrawn. For participants who lack capacity, a phone call or Zoom interview will be conducted with their family member/friend/carer/consultee.

### Eligibility criteria

#### Inclusion criteria

- Patients must be over the age of 65 years at the time of recruitment, admitted to an acute Elderly Care or General Medical Ward
- Patients to be identified when they are likely to be discharged within 48 h
- Patients need to be registered with a General Practitioner within the areas covered by the recruiting hospitals
- Informed written consent must be provided from patients with capacity OR personal consultees acting on behalf of patients lacking capacity to consent to participating in a research study

#### Exclusion criteria

- Patients lacking capacity and have no consultee to advise
- Patients that are transferred to other acute healthcare trusts (but excluding step down or intermediate care facilities)
- Patients who have a short life expectancy, due to a terminal illness
- Patients who are unable to read/speak/understand English

### Data collection

The baseline data to be collected and methodology will be similar to the original PRIME Study [43]. Baseline data to be collected from consenting participants (and participants consenting through their consultee) will include demographic (age, gender, ethnicity), clinical (discharge diagnosis, co-morbidities, renal function, electrolytes, hepatic function), and social indicators data (living arrangements and care package on discharge). Admission and discharge medication data (drug name, frequency, dosage) and use of compliance aids will be collected and coded using the WHO-ATC code [44].

Validated tools will be used to collect data on comorbidities, nutritional status, physical function, and cognitive function such as the Charlson Comorbidity Index (CCI) [45], Malnutrition Universal Screening Tool (MUST) [46] and Barthel Index [47].

Data collection will take place using an anonymised form designed to be scanned into an electronic database. At the point of data collection, each participant will be allocated a unique participant identification number (UPIN).

### Follow up data collection

A telephone interview and review of GP records after eight weeks’ post-discharge for every participant will be done using a standardised questionnaire to determine whether the patient has experienced MRH. Suspected ADRs, medication adherence, and primary/secondary care usage will be explored. MRH severity will be assessed using a modified Morimoto scale [48]. If an ADR is suspected, causality will be assessed using the Naranjo algorithm [49]. Medication adherence will be assessed using the Morisky scale [50].

Participants re-admitted to hospital within the study time frame will be reviewed prospectively to ascertain if MRH was the cause for re-admission. MRH occurrence will be the primary endpoint. Such patients will not re-enter the study as new participants.

### Statistical analysis plan

In the PRIME study [34], eight risk factors for medication-related harm (MRH) were identified: gender, age, past adverse drug reactions, antiplatelet drug, antidiabetic drug, living alone after discharge, sodium level (mmol/L) and total number of medicines at discharge. The univariate summaries of these variables will be provided and compared between participants in the control arm and participants in the intervention arm.

Baseline characteristics of participants included in the study will be analysed (using aggregated data and for data stratified by certain demographic characteristics) for descriptive purposes only.

Important variables known to be associated with MRH will also be described by randomisation groups. These variables will be considered in the analysis by fitting one multivariable logistic regression incorporating these variables to the data.

### Economic evaluation

The economic analysis will adopt the perspective of health and personal social services. Resources involved in delivering the interventions will be gathered prospectively. As in the original PRIME study, service use associated with incidents of medication-related harm will be collected retrospectively from three sources: phone interviews with participants, GP records and hospital records, including A&E attendance, hospital re-admissions, outpatient, GP, community care and social care. Costs of service use and the intervention will be based on validated national sources (PSSRU; National Reference costs) [51]. Costs will be compared between groups and modelled to provide national estimates [52].

### Process evaluation

Analysis of both quantitative and qualitative data collected from focus group interviews of researchers and service providers based on the need to investigate the acceptance and use of the study processes in both the control and the intervention arms will be done. The evaluation will explore the context of implementation of the proposed intervention in the four study sites. The focus of interviews will be based on previous studies of new ways of working to enhance medicines management that sought to include practitioner understanding in the adoption [53], and the development of recommendations to inform the scaling and sustainable roll-out of the study protocol in future practice.

### Declarations

#### Patient & public involvement

MRH predominately affects older people, as well as their carers. In the development of the study protocol, a Public and Community Engagement Committee (PCIE) consisting of an older person with links to the University of the Third Age (U3A) – a learning cooperative for those in later life, a carer for an older adult, and an expert patient. Their input contributed to developing the study protocol, and regular interactions with them will be undertaken during the study delivery.

## Dissemination

Results of this study will be presented at conferences such as the British Geriatrics Society (BGS) and articles published in peer reviewed journals. In addition, results will be disseminated to participants, patients and the public through the PCIE and relevant community groups.

The study participants, the KSS academic community, the national aging speciality group, and the care home community (via the Enabling Research in Care Homes (ENRICH) platform [54] will be notified of study updates with a newsletter every 4-months. The service evaluation aspect will provide knowledge of the enablers and barriers to scale up the study regionally and nationally. The National Adoption committee will work with Policy@Sussex and the ARC-KSS to continue to build awareness about the study and its findings.

## Supplementary Information

**Additional file 1: Appendix 1. **Formula for calculating patient risk of experiencing MRH within 8 weeks post hospital discharge. **Appendix 2.** Visual analogue of risk prediction tool. **Appendix 3.** Study participants flow chart.

## Data Availability

Data sharing is not applicable to this protocol as no datasets have been generated or analysed yet. The datasets generated and/or analysed during the study will be made available on request from the corresponding author.

## Abbreviations

ADE: Adverse Drug Event
AE: Adverse Event
ADR: Adverse Drug Reaction
AHSN: Academic Health Science Networks
ARC KSS: Applied Research Collaboration Kent, Surrey & Sussex
BGS: British Geriatrics Society
CI: Chief Investigator
DMS: Discharge Medicines Service
GCP: Good Clinical Practice
GP: General Practitioner
HWBH: Healthwatch Brighton and Hove
KSS: Kent, Surrey, and Sussex
MMP: Medicines Management Plan
MRH: Medication-Related Harm
NHS: National Health Service
PCIE: Public and Community Involvement and Engagement Committee
PI: Principal Investigator
PIS: Participant Information Sheet
PRIME: Prospective study to develop a model to stratify the RIsk of Medication-related harm in hospitalised Elderly patients
R&D: Research & Development
RCT: Randomised Control Trial
REC: Research Ethics Committee
RPM: Risk Prediction Model
RPT: Risk Prediction Tool
SOP: Standard Operating Procedure
U3A: University of the Third Age
UPIN: Unique Participant Identification Number
WHO: World Health Organisation
