# Supplementary material for: Implementation of a medicine management plan (MMP) to reduce medication-related harm (MRH) in older people post-hospital discharge: a randomised controlled trial
Source: BMC Geriatr. 2022 Nov 11;22:850. doi: 10.1186/s12877-022-03555-w (PMC9652884; doi:10.1186/s12877-022-03555-w)
Supplement: Supplementary file 1 — Additional file 1: Appendix 1. Formula for calculating patient risk of experiencing MRH within 8 weeks post hospital discharge. Appendix 2. Visual analogue of risk prediction tool. Appendix 3. Study participants flow chart. [file 12877_2022_3555_MOESM1_ESM.docx]

**Supplementary Information - Appendices**

***Appendix 1: Formula for calculating patient risk of experiencing MRH within 8 weeks post hospital discharge***


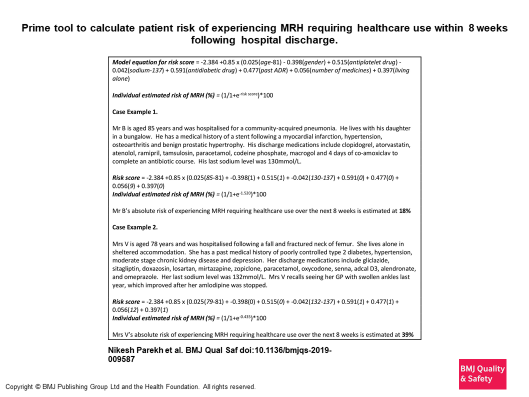


***Appendix 2: Visual analogue of risk prediction tool***


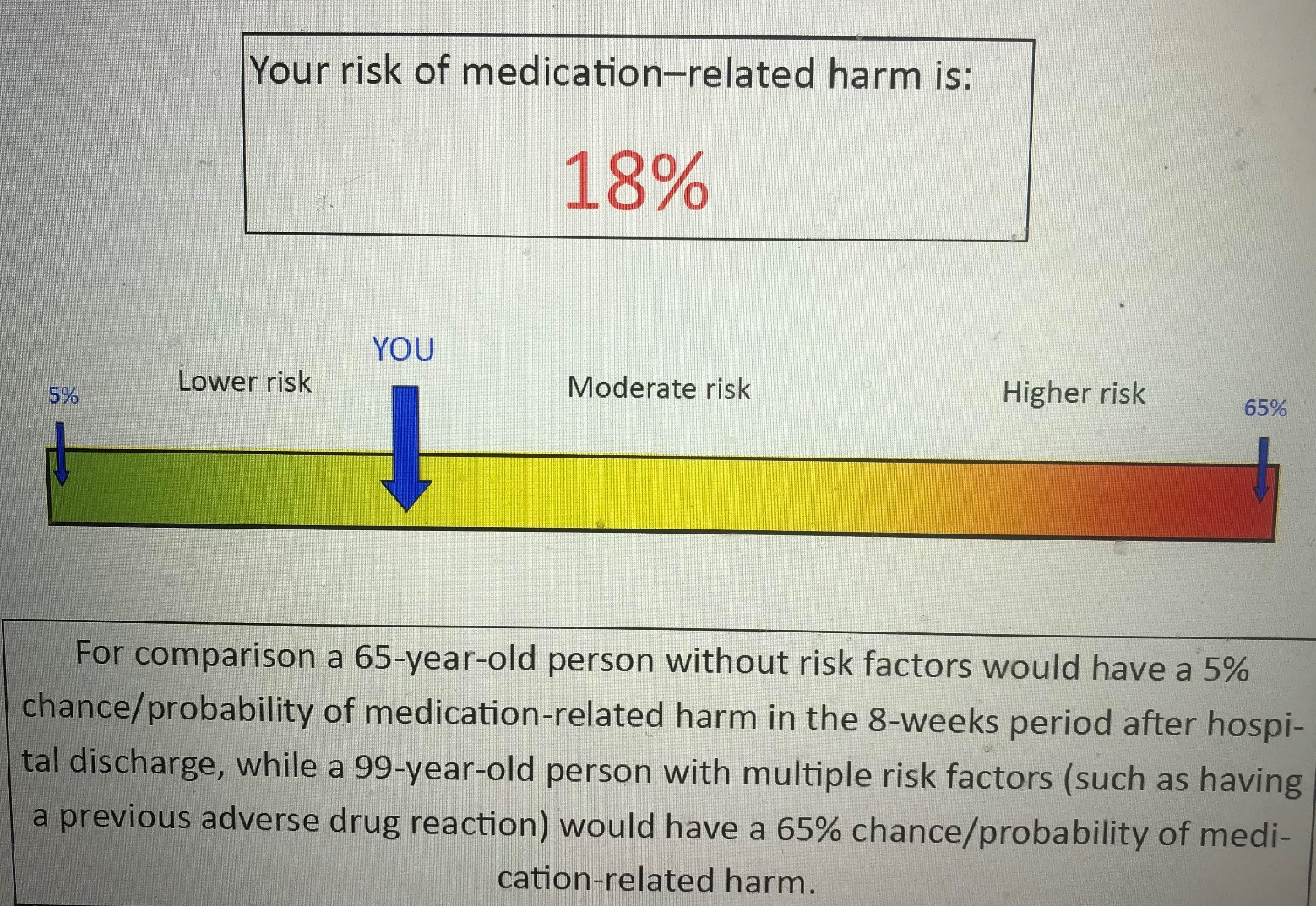


***Appendix 3: Study participants flow chart***

**Implementation of a risk prediction tool to reduce medication-related harm (MRH) in older people post-hospital discharge – Flow Chart.**

| Screening for eligible study participants by the research nurse (RN) |
| --- |

| Eligible participants will be approached by the RN and will be informed that they have an equal chance of being included in the intervention arm or the control arm (randomised) |
| --- |

| Eligible participants who agree to be randomised will be asked to provide consent to join the study |
| --- |

| Participants who provide consent will then be risk categorised using the risk prediction tool (RPT). |
| --- |

| Participants will then be randomised to the intervention or control arm |
| --- |

| Intervention arm (RPT + Medicine Management Plan + NHS DMS) |
| --- |

| Control arm (RPT + NHS DMS as usual standard of care) |
| --- |

| Study participants in both arms will be followed up at 8 weeks by the study pharmacist to verify occurrence of MRH |
| --- |
